# Imaging visuospatial memory in temporal lobe epilepsy—Results of an fMRI study

**DOI:** 10.1371/journal.pone.0264349

**Published:** 2022-02-22

**Authors:** Victor Schmidbauer, Karl-Heinz Nenning, Michelle Schwarz, Olivia Foesleitner, Gudrun Mayr-Geisl, Mehmet Salih Yildirim, Susanne Pirker, Doris Moser, Daniela Denk, Daniela Prayer, Karin Trimmel, Georg Langs, Christoph Baumgartner, Ekaterina Pataraia, Gregor Kasprian, Silvia Bonelli

**Affiliations:** 1 Department of Biomedical Imaging and Image-Guided Therapy, Medical University of Vienna, Vienna, Austria; 2 Department of Neurology, Medical University of Vienna, Vienna, Austria; 3 Department of Neurosurgery, Medical University of Vienna, Vienna, Austria; 4 General Hospital Hietzing with Neurological Center Rosenhuegel, Vienna, Austria; Medical University of Vienna, AUSTRIA

## Abstract

**Purpose:**

Impairment of cognitive functions is commonly observed in temporal lobe epilepsy (TLE). The aim of this study was to assess visuospatial memory functions and memory-related networks using an adapted version of *Roland’s Hometown Walking* (RHWT) functional MRI (fMRI) task in patients with TLE.

**Methods:**

We used fMRI to study activation patterns based on a visuospatial memory paradigm in 32 TLE patients (9 right; 23 left) and also within subgroups of lesional and non-lesional TLE. To test for performance, a correlational analysis of fMRI activation patterns and out-of-scanner neuropsychological visuospatial memory testing was performed. Additionally, we assessed memory-related networks using functional connectivity (FC).

**Results:**

Greater contralateral than ipsilateral mesiotemporal (parahippocampal gyrus/hippocampus) activation was observed in left (n = 23)/right (n = 9) TLE. In lesional left TLE (n = 17), significant activations were seen in right more than left mesiotemporal areas (parahippocampal gyrus), while non-lesional left TLE patients (n = 6) showed significant bilateral (left>right) activations in mesiotemporal structures (parahippocampal gyrus). In left TLE, visuospatial cognitive testing correlated with fMRI activations in left (parahippocampal gyrus) and right mesiotemporal structures (hippocampus), characterized by greater fMRI activation being associated with better memory scores. In right TLE, higher scores in visuospatial memory testing were associated with greater fMRI activations in left and right insular regions. FC patterns of memory-related networks differ in right and left TLE.

**Conclusion:**

While TLE in general leads to asymmetrical mesiotemporal activation, lesion-induced and non-lesional TLE patients reveal different memory fMRI activation patterns. In right TLE, insular regions try to compensate for impaired right mesiotemporal structures during the performance of visuospatial tasks. Underlying functional visuospatial memory networks differ in right and left TLE.

## Introduction

Temporal lobe epilepsy (TLE) is the most frequent type of focal epilepsy [1] and remains drug resistant in 30% of the cases [2].

If remission of temporal lobe seizures is not achievable by antiepileptic drug treatment, epilepsy-surgery might be an alternative treatment option [1, 3]. A detailed pre-surgical assessment using prolonged video-electroencephalography (EEG) monitoring, neuropsychological and neuropsychiatric evaluation, structural magnetic resonance imaging (MRI), and functional MRI (fMRI) provide important information on the localization of the epileptogenic zone, which has to be removed and essential brain regions [4, 5], which have to be spared during epilepsy-surgery. Hence, precise prediction of the individual outcome after epilepsy-surgery is the ultimate goal of clinical neuroimaging.

Jokeit et al. used fMRI and an adapted version of the *Roland’s Hometown Walking task* (RHWT) [6], in order to investigate hemispheric activation asymmetries in TLE patients [7]. Subjects mentally navigated through their hometown and tried to recall as many details as possible [6, 7]. In healthy subjects, a symmetrical, bilateral activation pattern was observed in mesiotemporal regions. In contrast, TLE patients showed reduced fMRI activations on the side of the seizure focus [7]. The same paradigm provided valuable results for the prediction of visual memory impairment after right-sided temporal lobe resection [8].

Several studies have identified alterations in functional connectivity (FC) among TLE patients [9, 10]. Based on a spatial fMRI task, Doucet et al. demonstrated that left and right TLE patients showed different FC patterns [11, 12]. Adapted versions of the RHWT have been considered, in order to detect differences of fMRI activation patterns in left and right TLE compared to healthy subjects [13]. However, studies using fMRI to investigate memory-related networks in patients with TLE are still scarce.

In this study, we retrospectively analyzed memory fMRI data that were obtained using an adapted version of the RHWT, to assess visual memory fMRI activation patterns in left and right TLE patients and subgroups comparing lesional versus non-lesional TLE.

To test for performance, memory fMRI imaging results were correlated with the results of out-of-scanner neuropsychological testings.

In order to assess functional networks underlying visuospatial memory and how these differ between different TLE groups, task-based FC analysis was performed. As it is currently discussed that the default mode network (DMN) may have a key role during cognitive processing [14], also mesiotemporal integration into anterior and posterior DMN components was analyzed.

This study aims to supply additional information beyond the data provided by Jokeit et al. [7], by investigating visual memory-related activation patterns in TLE patients with different underlying pathologies and particularly non-lesional TLE patients. Furthermore, using RHWT-based functional connectivity analysis the present study pursues to identify memory-related networks subserving this task and relevant reorganization mechanisms.

## Materials and methods

### Ethical approval

The Ethics Commission of the Medical University of Vienna approved the protocol of this study, which was performed in accordance with the Declaration of Helsinki. All patients gave written informed consent prior to fMRI and agreed to the scientific use of the acquired data.

### Study cohort

Between 01/2013 and 09/2017, 32 patients (Table 1) with medically intractable TLE underwent memory fMRI, using an adapted version of the RHWT at the Department of Neuroradiology of a tertiary care hospital. Patients were referred for neuroradiological assessment by several Departments of Neurology located in Vienna including two tertiary care centers. All patients underwent (video-)EEG monitoring, structural MRI, and language and memory fMRI. (Video-)EEG monitoring revealed a right seizure onset in 9/32 and a left seizure onset in 23/32 patients. Structural MRI revealed an underlying pathology in 17/23 left and 7/9 right TLE patients. Patients were classified as MRI negative (n = 8), if no structural alteration was detected on MRI. Additionally, in 12 patients results of comprehensive neuropsychological testing including assessment of visuospatial memory functions were available.

**Table 1:** Clinical characteristics.

Patient	Pathology/Condition	Hand	Age	Duration	Sex	Language	Seizure Onset^a^
1^b^	HS	L	41y	2y	M	L	R
2^b^	HS	L	54y	19y	M	L	R
3^b^	HS	L	34y	6y	M	L	R
4^b^	GAD+ Encephalitis	R	37y	3y	F	L	R
5^b^	MCD	L	19y	6y	M	L	R
6^b^	Ganglioglioma (16.5x12.8)^f^	R	39y	3y	F	L	R
7^b^	MCD	R	25y	2y	M	Bilateral	R
8^c^	HS	R	22y	6y	F	L	L
9^c^	HS	R	26y	22y	M	L	L
10^c^	HS	R	23y	6y	F	L	L
11^c^	HS	R	9y	4y	F	L	L
12^c^	HS	R	58y	52y	M	L	L
13^c^	HS	R	16y	5y	F	L	L
14^c^	HS	R	54y	35y	M	L	L
15^c^	HS	R	16y	16y	M	L	L
16^c^	Oligoastrocytoma (64.3x49.3)^f^	R	46y	1m	M	L	L
17^c^	Astrocytoma (72.9x41.4)^f^	R	29y	2y	M	L	L
18^c^	Astrocytoma (75.7x41.4)^f^	R	41y	2y	M	L	L
19^c^	Cavernoma	R	55y	1m	M	L	L
20^c^	Cavernoma	R	46y	3y	M	Bilateral	L
21^c^	MCD	L	42y	16y	M	R	L
22^c^	Ganglioglioma (38.6x30.7)^f^	R	18y	5y	M	L	L
23^c^	DNET/Astrocytoma (34.1x20.3)^f^	R	23y	4m	F	L	L
24^c^	Glioma/DNET/Ganglioglioma (32.8x14.5)^f^	R	26y	1y	F	L	L
25^d^	MRI negative	R	48y	3y	F	Bilateral	R
26^d^	MRI negative	R	51y	11y	M	L	R
27^e^	MRI negative	R	33y	4y	F	L	L
28^e^	MRI negative	R	35y	2y	M	L	L
29^e^	MRI negative	R	56y	1y	M	L	L
30^e^	MRI negative	L	31y	24y	F	Bilateral	L
31^e^	MRI negative	R	29y	24y	M	L	L
32^e^	MRI negative	X	43y	15y	F	L	L

^a^ Based on video-EEG monitoring and EEG findings

^b^ Lesional right TLE

^c^ Lesional left TLE

^d^ Non-lesional right TLE

^e^ Non-lesional left TLE

^f^ Maximum diameters (mm) of (temporal lobe) tumor-related tissue alterations assessed on T2-weighted contrasts (axial plane)

Age: Age at data acquisition

Duration: Period between age at first diagnosis and data acquisition

Hand: Handedness

Language: Language lateralization assessed by clinical language fMRI

Seizure Onset: Hemisphere in which the seizure is generated

DNET: Dysembryoplastic neuroepithelial tumor

F: Female

GAD: Glutamic acid decarboxylase

HS: Hippocampal sclerosis

L: Left

m: Month

M: Male

MCD: Mild malformation of cortical development

MRI: Magnetic resonance imaging

R: Right

X: Unknown

y: Year

### Groups

Subjects were divided into groups based on the side of the seizure origin and evidence of mesiotemporal pathology:

1. fMRI group activations were compared in left TLE (n = 23) and right TLE (n = 9) (*main activation analysis*). In addition, subgroup analysis was performed in lesional left TLE (n = 17), lesional right TLE (n = 7), and left MRI negative TLE (n = 6) (*subgroup activation analysis*). No subgroup analysis was possible in right MRI negative TLE patients, due to the small sample size (n = 2). Demographic information of the different groups is given in Table 2. Descriptive statistics were performed using SPSS Statistics for Macintosh, Version 25.0 (IBM Corp, 2017).
2. For correlational analysis with out-of-scanner neuropsychological test results (available in 12 patients) (S1 Table), subjects were grouped into lesional left (n = 6) and lesional right TLE (n = 6).
3. FC was analyzed separately in left (n = 23) and right (n = 9) TLE (*main functional connectivity analysis*). In addition, FC subgroup analysis was performed in lesional left TLE (n = 17), lesional right TLE (n = 7), and left MRI negative TLE (n = 6) (*subgroup functional connectivity analysis*).

**Table 2:** Demographic data of the groups.

	Left TLE	Right TLE	Left TLE–Lesional	Right TLE–Lesional	Left TLE–MRI negative
**Subjects**	n = 23	n = 9	n = 17	n = 7	n = 6
**Sex**	9/14 (F/M)	3/6 (F/M)	6/11 (F/M)	2/5 (F/M)	3/3 (F/M)
**Handedness**	20/2/1 (R/L/X)	5/4 (R/L)	16/1 (R/L)	3/4 (R/L)	4/1/1 (R/L/X)
**Median age** *	31 (9–58)	39 (19–54)	26 (9–58)	37 (19–54)	34 (29–56)
**Median duration** *	5 (0.083–52)	3 (2–19)	5 (0.083–52)	3 (2–19)	9.5 (1–24)

* Data represented in years complemented by range (in parentheses)

Median age: Median age at data acquisition

Median duration: Median period between age at first diagnosis and data acquisition

F: Female

L: Left

M: Male

MRI: Magnetic resonance imaging

R: Right

TLE: Temporal lobe epilepsy

X: Unknown

### MRI data acquisition

Imaging data were acquired using a 3 Tesla MRI scanner (Philips Medical System, Best, Netherlands) equipped with a 12-channel head coil. A high resolutional structural T1-image [repetition time (TR)/echo time (TE) = 8/3 ms, flip angle: 8°, matrix: 320x320x195, voxel size: 0.75x0.75x1 mm] was acquired. An echo planar imaging sequence was used to acquire the fMRI data (TR/TE = 3000/35 ms, flip angle: 90°, matrix: 128x128x32, voxel size: 1.8x1.8x4 mm) with a duration of 5 minutes.

### fMRI paradigm

For this study, an adapted version of the RHWT was used [6, 7]. Patients were instructed to mentally navigate in a familiar environment and to remember as many details as possible. The task was performed in block design. Activation phases (five cycles) and resting phases (five cycles) lasted for 30 seconds each, for a total duration of five minutes. During the activation phase of the task, subjects were instructed to perform the mental navigation. During the resting phase, patients were instructed to stop mental navigation at the point reached. The task always started with the resting phase, followed by an activation phase. Subjects were asked to keep their eyes closed during the task, in order to minimize visual arousal. Via an intercommunication system, information for starting and stopping the mental navigation was given throughout the process of data acquisition. Patients had to continue mental navigation from that point, where they had stopped in the previous phase. During scanning, task activation was monitored using an online processing tool, which allowed the assessment of incorrect performance of the paradigm. In such cases, the task was explained again and repeated as described above.

### Neuropsychological assessment

Neuropsychological scores for spatial learning and three-dimensional learning, obtained during routine pre-surgical evaluation were used for correlation with fMRI data in this study.

Data of the following neuropsychological tests were used for correlational analysis:

○ *Mosaik test*: Coloured building blocks are used to copy a given pattern. The test determines non-verbal conceptualization, visual perception and organization, visual motor coordination, and spatial imagination [15, 16].
○ *Maze test*: Subjects must draw the right path through a maze, which tests higher cognitive functions and predictive planning [17, 18].
○ *LGT-3 test*: Subjects have to memorize phone numbers, vocabulary, details of a text, city maps, various objects, and symbols. The task determines verbal and spatial skills [19].
○ *LPS*-7 *test*: In several lines, four letters or numbers are printed in different rotations. A fifth character is mirrored and should be recognized and crossed out. The test evaluates spatial perception [20].

### Data preprocessing, activation analysis, and correlational analysis

Image preprocessing and data analysis was performed using statistical parametric mapping (SPM) 12 (http://www.fil.ion.ucl.ac.uk/spm/software/spm12/) and MATLAB R2017a.

Standard fMRI preprocessing was employed. First, fMRI data were motion corrected by realigning each volume to the mean as a reference, and subsequently, co-registered to the structural T1-weighted volume. The T1-weighted images were spatially normalized to the Montreal Neurological Institute (MNI) template space (resolution: 1x1x1 mm), in order to generate comparable data across subjects. Finally, the same normalization transformation was applied to the co-registered functional data, followed by a spatial smoothing with a Gaussian kernel of 8 mm full-width at half maximum.

A two-level random-effect analysis was employed for all imaging data. At the first level, condition specific effects for each subject were estimated with the general linear model (GLM) [21]. For each patient, task-specific effects were estimated via the contrast task against rest. The obtained contrast images were used for the second level group analysis.

At the second level, for each group, a within-group analysis was performed using a one-sample t-test.

In order to test for correlations between areas of fMRI activation and subject’s performance on the *Mosaik-*, *Maze-*, *LGT-3-*, and *LPS test* simple regression analyses were performed over the whole brain.

fMRI activations were reported at a threshold with a significance level of *p*≤0.001 uncorrected/*p*<0.05 family-wise error (FWE) corrected. Only activations with a cluster size of ≥10 were reported. Due to the small sample size, also non-significant trends were reported for correlational analysis. Special attention was paid to mesiotemporal regions.

### Functional connectivity analysis

For FC analysis, data were preprocessed using CONN FC toolbox (18.b) (https://web.conn-toolbox.org). CONN’s “default preprocessing pipeline”was used, involving the following steps: structural segmentation (grey matter, white matter, CSF) and MNI normalization; functional realignment and unwarp (subjects motion estimation and correction); co-registration to the structural image; slice-timing correction; functional outlier detection (global signal *z* threshold: 3; subjects motion threshold: 0.9 mm); functional smoothing (Gaussian kernel filter of 8 mm full-width at half maximum) [22, 23].

Subsequently, a denoising process was performed by applying a band-pass filter (0.008–0.09 Hz), in order to decrease noise and low-frequency drifts.

A region of interest (ROI) to ROI analysis was performed. For this purpose, correlation maps were created for each seed-region in order to calculate significant connectivities between different ROIs [22, 23]. By applying a GLM [21] and bivariate correlation analysis weighted for haemodynamic response function the corresponding correlations were estimated. Fisher’s transformation was applied for all bivariate correlation analysis calculated *z*-values and correlation coefficients were converted into standard scores. Hence, high *z*-values between ROIs represent positive correlations and low *z*-values indicate negative correlations [22, 23].

ROIs were defined by means of CONN’s standard atlas: the Harvard-Oxford atlas for cortical and subcortical regions and the Automated Anatomical Labeling atlas for cerebellar regions [22].

ROI to ROI analysis was performed for each group separately for the active part of the task. Based on previous findings of group activation peaks, the following seed-regions were chosen: right and left parahippocampal gyrus, hippocampus, and insular cortex. Results were corrected with a false discovery rate (FDR) of 5%. Only the most significant connectivities for each region were reported.

Additionally, an independent component analysis (ICA) was performed which allows to extract specific networks for separate assessment [24]. Due to the potential role of the DMN during cognitive tasks [14], mesiotemporal integration into anterior and posterior DMN components was analyzed in each group. CONN uses a group ICA with Back-Projection according to the method proposed by Calhoun et al. [24]. Only the highest significant mesiotemporal values were reported (threshold level at *p*≤0.001 uncorrected).

## Results

### Functional MRI–activation analysis

#### Visuospatial memory fMRI activation in left TLE (main activation analysis)

**Fig 1:**
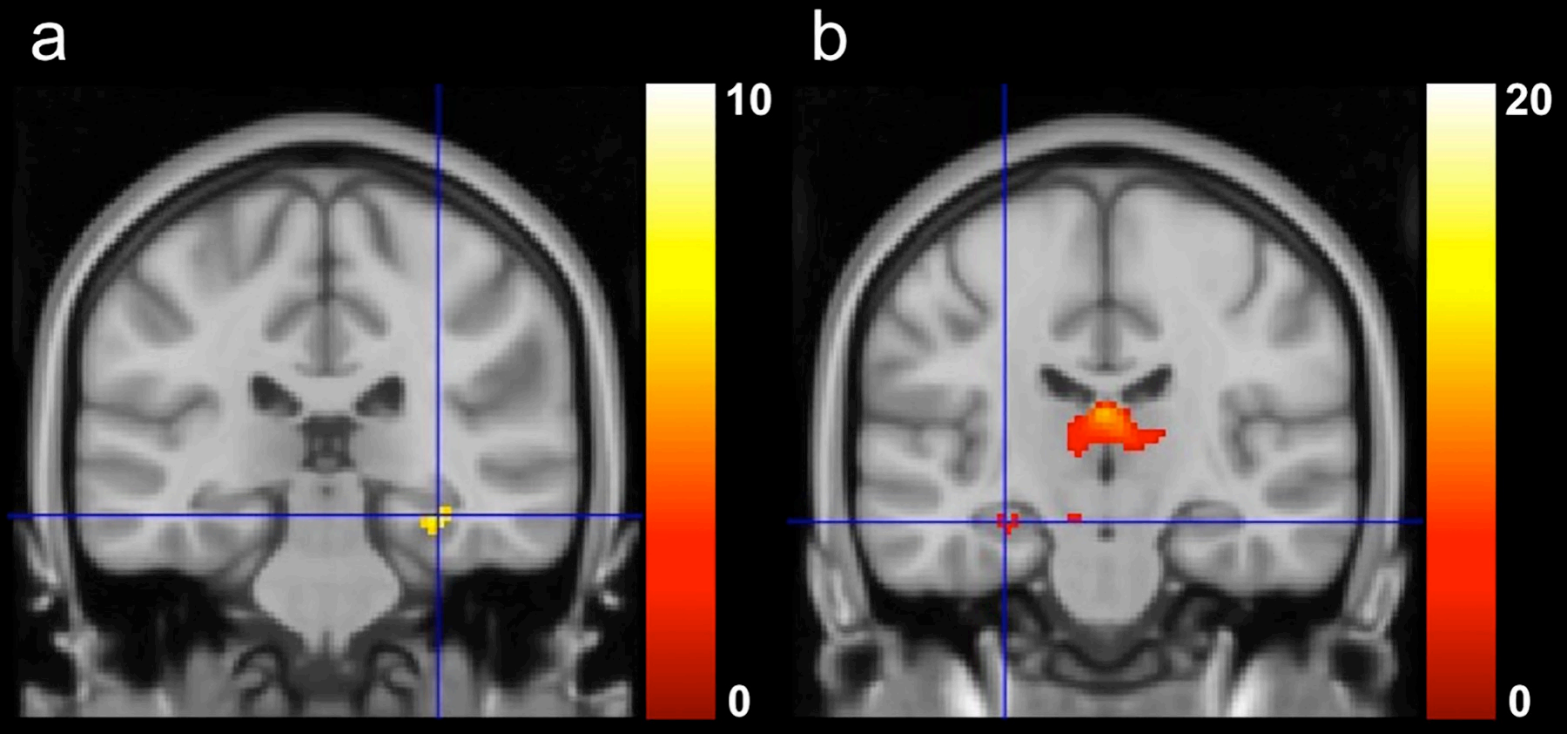
Visuospatial memory fMRI activation in left and right TLE. In left TLE (a), significant fMRI activations were observed in right [cross-hair in a: parahippocampal gyrus (coordinates: 32–30–14), posterior division; *p*(FWE) = 0.007] mesiotemporal regions. In contrast, right TLE patients (b) showed greater fMRI activation in left mesiotemporal regions [cross-hair in b: hippocampus (coordinates: -28–20–16); *p*(uncorrected)≤0.001]. Images are displayed at a threshold level at *p*<0.05 (FWE) (a)/*p*≤0.001 (uncorrected) (b) (left: left hemisphere; right: right hemisphere).

Highly significant activations were found in right mesiotemporal structures [parahippocampal gyrus, posterior division: *p*(uncorrected)≤0.001, *p*(FWE) = 0.007, *z* = 5.17] (Fig 1), but also within extratemporal areas (Table 3).

**Table 3:** fMRI activation peaks in left and right TLE.

Group	Anatomical Region	Coordinates	*z*-score	*p*-value*	FWE
**Left TLE (n = 23)**	Right intracalcarine cortex	16–62 12	6.24	≤0.001	≤0.001
	Left precuneus cortex	-16–58 12	5.60	≤0.001	0.001
	Right lateral occipital cortex, superior division	38–76 32	5.39	≤0.001	0.003
	Right parahippocampal gyrus, posterior division	32–30–14	5.17	≤0.001	0.007
	Left lateral occipital cortex	-30–78 30	5.02	≤0.001	0.013
	Left superior frontal gyrus	-24 6 56	4.99	≤0.001	0.015
**Right TLE (n = 9)**	Left inferior temporal gyrus, temporooccipital part	-48–50–12	5.49	≤0.001	0.003
	Left supramarginal gyrus, anterior division	-42–40 42	4.91	≤0.001	
	Left superior frontal gyrus	-20–10 64	4.86	≤0.001	
	Left lateral occipital cortex, superior division	-44–66 22	4.50	≤0.001	
	Left middle temporal gyrus, temporooccipital part	-50–56 2	4.39	≤0.001	
	Left cingulate gyrus	-14–36 34	4.17	≤0.001	
	Left precuneus cortex	-22–54 8	4.09	≤0.001	
	Right superior frontal gyrus	28 6 64	4.04	≤0.001	
	Left hippocampus	-28–20–16	3.37	≤0.001	

* Uncorrected *p*-values

FWE: Family-wise error corrected

MRI: Magnetic resonance imaging

TLE: Temporal lobe epilepsy

#### Visuospatial memory fMRI activation in right TLE (main activation analysis)

There were significant activations in left mesiotemporal structures [hippocampus: *p*(uncorrected)≤0.001, *z* = 3.37] (Fig 1), but also within extratemporal areas (Table 3).

#### Visuospatial memory fMRI activation in lesional left TLE (subgroup activation analysis)

Greater activations were found in right [parahippocampal gyrus, posterior division: *p*(uncorrected)≤0.001, *z* = 4.49] than left [parahippocampal gyrus, posterior division: *p*(uncorrected)≤0.001, *z* = 3.78] mesiotemporal structures, but also within extratemporal areas (Table 4).

**Table 4:** fMRI activation peaks in lesional and MRI negative TLE.

Group	Anatomical Region	Coordinates	*z*-score	*p*-value*	FWE
**Lesional left TLE (n = 17)**	Right intracalcarine cortex	16–62 10	5.42	≤0.001	0.004
	Right lateral occipital cortex, superior division	40–76 32	4.90	≤0.001	0.030
	Left paracingulate gyrus	-8 12 42	4.69	≤0.001	
	Left precuneus cortex	-8–62 10	4.63	≤0.001	
	Left frontal lobe	-36 40–12	4.50	≤0.001	
	Right parahippocampal gyrus, posterior division	32–30–14	4.49	≤0.001	
	Left lateral occipital cortex, superior division	-30–76 32	4.28	≤0.001	
	Left parahippocampal gyrus, posterior division	-18–28–18	3.78	≤0.001	
**Lesional right TLE (n = 7)**	Left inferior temporal gyrus, temporooccipital part	-46–50–12	4.78	≤0.001	
	Left supramarginal gyrus, posterior division	-60–44 20	4.75	≤0.001	
	Left insular cortex	-30 16 4	4.58	≤0.001	
	Left lateral occipital cortex, superior division	-38–66–30	4.53	≤0.001	
	Right frontal pole	34 38 34	4.53	≤0.001	
	Left superior parietal pole	-32–52 52	4.40	≤0.001	
	Right occipital pole	20–98 2	4.31	≤0.001	
	Right superior frontal gyrus	18–2 70	4.15	≤0.001	
	Right middle frontal gyrus	32 2 56	4.08	≤0.001	
**Left MRI negative TLE (n = 6)**	Left temporal fusiform cortex	-38–28–18	4.56	≤0.001	
	Left middle frontal gyrus	-28 2 44	4.47	≤0.001	
	Right precentral gyrus	46 6 32	4.39	≤0.001	
	Left lateral occipital cortex, superior division	-36–72 22	4.11	≤0.001	
	Left supracalcarine cortex	-8–66 16	4.03	≤0.001	
	Left parahippocampal gyrus, posterior division	-28–26–26	3.83	≤0.001	
	Right parahippocampal gyrus, posterior division	18–24–20	3.73	≤0.001	

* Uncorrected *p*-values

FWE: Family-wise error corrected

MRI: Magnetic resonance imaging

TLE: Temporal lobe epilepsy

#### Visuospatial memory fMRI activation in lesional right TLE (subgroup activation analysis)

No significant activations were found in mesiotemporal regions, but mainly in the left inferior temporal and supramarginal gyrus, the left insular and occipital cortex, and right frontal regions (Table 4).

#### Visuospatial memory fMRI activation in left MRI negative TLE (subgroup activation analysis)

Significant activations were observed bilaterally in left [parahippocampal gyrus, posterior division: *p*(uncorrected)≤0.001, *z* = 3.83] and right [parahippocampal gyrus, posterior division: *p*(uncorrected)≤0.001, *z* = 3.73] mesiotemporal structures. Additionally, significant activations were seen in the left fusiform gyrus, the left middle frontal gyrus, and the occipital cortex (Table 4).

### Correlational analysis with out-of-scanner neuropsychological test results

#### Correlational analyses in lesional left TLE

Significant correlations were observed exclusively in left mesiotemporal areas [parahippocampal gyrus, posterior division: *p*(uncorrected)≤0.001, *z* = 4.01], characterized by greater fMRI activation being associated with better performance during the *Mosaik test*. Better performance during the *LGT-3-*, *LPS-7-*, *and Maze test* was associated with greater fMRI activations in right mesiotemporal regions (Table 5).

**Table 5:** Correlational analyses with neuropsychological data.

Group	Test	Anatomical Region	Coordinates	*z*-score	*p*-value*
**Lesional left-sided TLE (n = 6)**	*Mosaik test*	Left parahippocampal gyrus, posterior division	-26–40–12	4.01	≤0.001
	*Mosaik test*	Right hippocampus	22–12–22	2.68	0.004
	*Maze test*	Right hippocampus	32–10–16	3.14	0.001
	*LPS-7 test*	Right parahippocampal gyrus, posterior division	32–32–10	2.88	0.002
	*LGT-3 test*	Right parahippocampal gyrus, posterior division	34–32–10	3.08	0.001
**Lesional right-sided TLE (n = 6)**	*LGT-3 test*	Left insular cortex	-32 16 2	4.17	≤0.001
	*LPS-7 test*	Left insular cortex	-26 26 0	4.00	≤0.001
	*Mosaik test*	Right frontal operculum	48 14–4	4.77	≤0.001
	*Mosaik test*	Left insular cortex	-28 26–2	4.48	≤0.001
	*Mosaik test*	Right insular cortex	40 16–4	4.28	≤0.001

* Uncorrected *p*-values

TLE: Temporal lobe epilepsy

#### Correlational analyses in lesional right TLE

No significant correlations between mesiotemporal fMRI activations and out-of-scanner neuropsychological testings were found. Instead, significant correlations were observed between fMRI activation in the left insular cortex and scores of the *LGT-3-*, *LPS-7-*, *and Mosaik test* characterized by greater activation being correlated with better performance. Furthermore, significant correlations were found between fMRI activations in the right insular cortex and frontal operculum and better performance during the *Mosaik test* (Table 5). No significant correlations were seen for the *Maze test*.

### Functional connectivity analysis

#### ROI to ROI analysis

For better characterization of the underlying visuospatial memory networks, seed-based FC analysis was performed.

**Fig 2:**
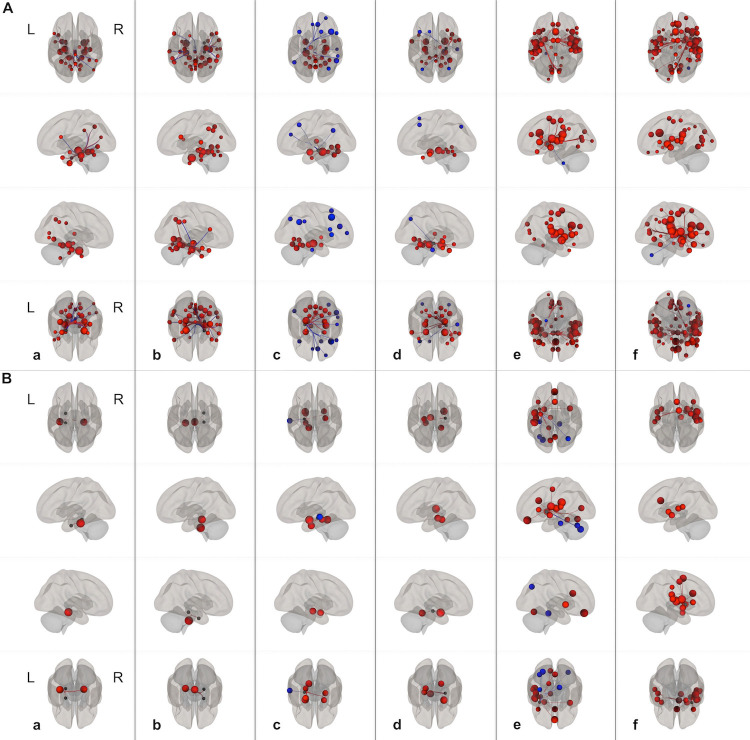
Functional connectivity. A: Functional connectivity in left TLE. B: Functional connectivity in right TLE. Seed-regions are indicated by the columns (a, b, c, d, e, f). a: left parahippocampus; b: right parahippocampus; c: left hippocampus; d: right hippocampus; e: left insula; f: right insula. Upper row: superior view; second row: left view; third row: right view; bottom row: inferior view. Compared to subjects with left TLE, right TLE patients show reduced FC of left-sided and right-sided mesiotemporal regions within temporal and to extratemporal brain areas. As opposed to mesiotemporal areas, the FC pattern of left and right insular regions appears less affected. In general, right TLE patients show pronounced FC alterations compared to left TLE patients. Images are displayed at a threshold level at *p*<0.05 (FDR). L: left hemisphere; R: right hemisphere.

Fig 2 gives an overview of the connectivity patterns in left and right TLE.

*Left TLE (main functional connectivity analysis)*. In left TLE, significant connectivities were observed between the left/right parahippocampal gyrus and contralateral temporal areas and to extratemporal regions. However, there was greater connectivity between right parahippocampal regions to frontal areas as compared to the contralateral side. There was strong connectivity between the left/right hippocampus and contralateral temporal areas and to extratemporal regions. Negative correlations (anticorrelations) were found, predominantly, between the left hippocampus and frontal lobe areas. Both left and right insular cortex revealed widespread connectivity to ipsilateral and contralateral frontal, temporal, parietal, and occipital areas. Detailed information is given in the S1 Text.

*Right TLE (main functional connectivity analysis)*. In right TLE, there were significant connectivities between ipsilateral and contralateral mesiotemporal areas. However, there was poor connectivity between mesiotemporal regions and extratemporal areas. Negative correlations (anticorrelations) were observed within left temporal regions. Both left and right insular cortex revealed widespread connectivity to ipsilateral and contralateral brain regions. Detailed information is given in the S2 Text.

*Lesional left TLE (subgroup functional connectivity analysis)*. In lesional left TLE, significant connectivities were observed between the left and right parahippocampal gyrus and contralateral mesiotemporal areas. There were considerable connectivities between mesiotemporal regions and parietal areas. However, as opposed to the right parahippocampal gyrus, the left parahippicampal gyrus showed reduced connectivity to extratemporal regions. Negative correlations were observed, predominantly, between the left hippocampus and frontal areas. Both left and right insular cortex revealed widespread connectivity to ipsilateral and contralateral frontal, temporal, parietal, and occipital areas. Detailed information is given in the S3 Text.

*Lesional right TLE (subgroup functional connectivity analysis)*. In lesional right TLE, significant connectivities were observed between left mesiotemporal areas and extratemporal regions. Negative correlations were observed within left temporal areas. Both left and right insular cortex revealed strong connectivity to temporal regions. Detailed information is given in the S4 Text.

*Left MRI negative TLE (subgroup functional connectivity analysis)*. In non-lesional left TLE, significant connectivities were observed within mesiotemporal regions, primarily originating from the right hippocampus. As opposed to the left insular cortex, right insular areas revealed a more complex connectivity pattern. Detailed information is given in the S5 Text.

#### Independent component analysis

In order to specifically assess how mesiotemporal structures are linked to the DMN and how this may secondarely alter task-positive cognitive networks ICA was performed.

**Fig 3:**
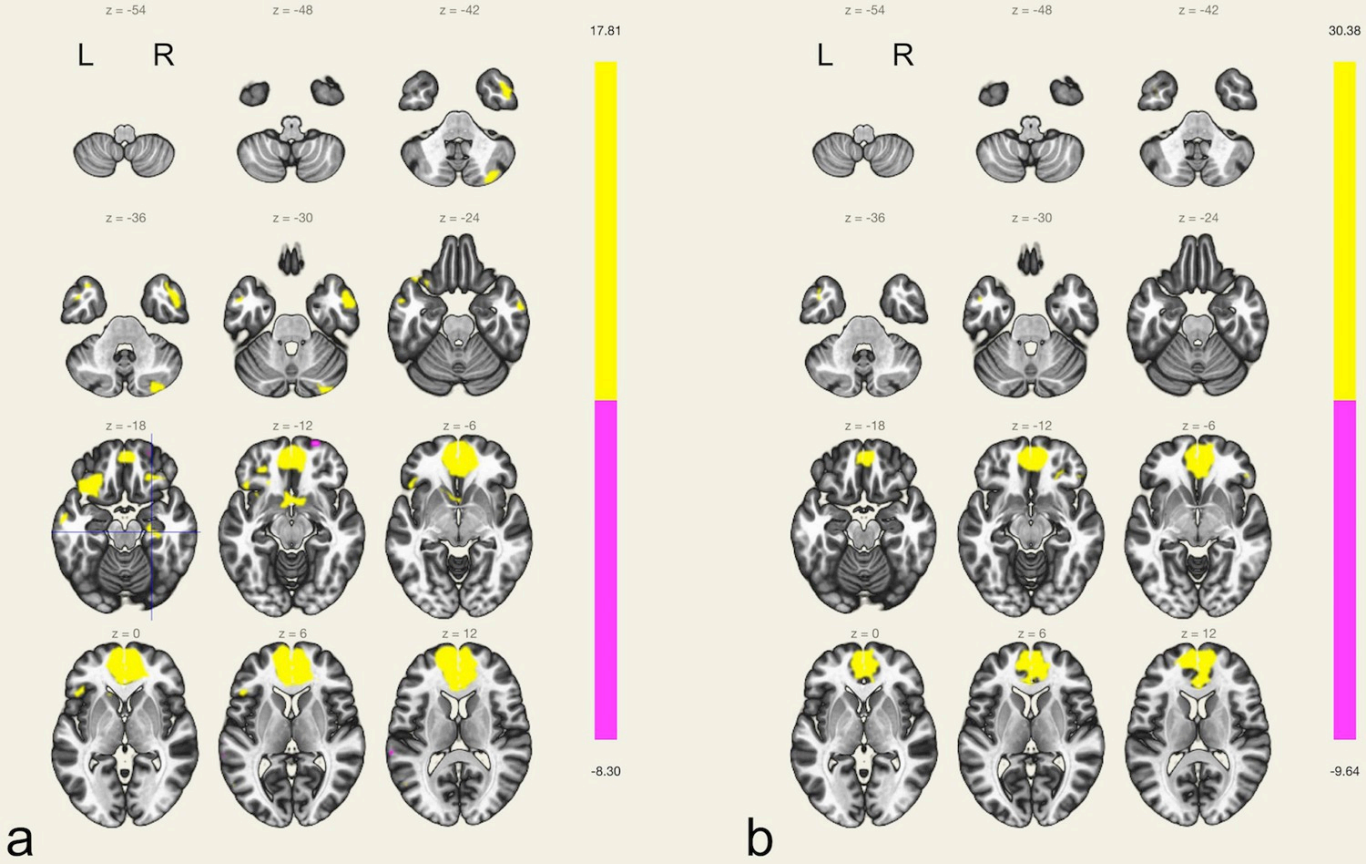
Hippocampal integration into the DMN. 3a: Left TLE: Integration of the right hippocampus (crosshair) into the anterior DMN. 3b: Right TLE: No mesiotemporal structures are integrated into the anterior DMN. Images are displayed at a threshold level at *p*≤0.001 (uncorrected). L: left hemisphere; R: right hemisphere.

*Left TLE (main functional connectivity analysis)*. In left TLE, there was integration of both left and right parahippocampal gyrus, posterior division [left: p(uncorrected)≤0.001, z = 6.32; right: p(uncorrected)≤0.001, z = 6.26] within the posterior DMN, while there was only right mesiotemporal integration [hippocampus: p(uncorrected)≤0.001, z = 4.45] within the anterior DMN (Fig 3).

*Right TLE (main functional connectivity analysis)*. In right TLE, there was integration of left mesiotemporal structures [parahippocampal gyrus, posterior division: *p*(uncorrected)≤0.001, *z* = 4.25] within the posterior DMN. However, with regard to the anterior DMN, no mesiotemporal integration was observed (Fig 3).

*Lesional left TLE (subgroup functional connectivity analysis)*. In lesional left TLE, integration of both left and right hippocampus [left: *p*(uncorrected)≤0.001, *z* = 4.263; right: *p*(uncorrected)≤0.001, *z* = 4.33] within the posterior DMN was observed. As opposed to that, there was only right mesiotemporal integration [right hippocampus: *p*(uncorrected)≤0.001, *z* = 4.214] within the anterior DMN.

*Lesional right TLE (subgroup functional connectivity analysis)*. In lesional right TLE, integration of left mesiotemporal structures [left parahippocampal gyrus, posterior division: *p*(uncorrected)≤0.001, *z* = 7.247] within the posterior DMN was found. However, with regard to the anterior DMN, no mesiotemporal integration was observed.

*Left MRI negative TLE (subgroup functional connectivity analysis)*. In non-lesional TLE, ICA did not reveal mesiotemporal integration within the anterior or posterior DMN.

## Discussion

### Summary of main findings

In this study, we retrospectively analyzed memory fMRI data based on an adapted version of the RHWT in order to investigate visuospatial memory function in TLE patients. We used a paradigm that required different components of memory functions, however, visuospatial memory components were primarily relevant for performing this task adequately.

In all patients, we demonstrated activations within frontoparietal, temporal, or occipital areas as part of the visuospatial memory network. In (lesional) left and right TLE relatively greater activation was seen in contralateral mesiotemporal structures, while in subjects with non-lesional left TLE, bilateral mesiotemporal activations were observed.

Furthermore, we showed that in patients with lesional left TLE, higher scoring in visuospatial neuropsychological testing was correlated with fMRI activations exclusively within right and left mesiotemporal structures. In lesional right TLE, a significant correlation was observed within extratemporal regions in both, left and right insular regions.

Connectivity analysis revealed reduced mesiotemporal FC in right compared to left TLE patients.

### Visual memory fMRI in TLE

fMRI is considered a promising pre-operative tool for assessing the lateralization and localization of memory function and to estimate the post-surgical risk of impairment [7, 8, 25, 26]. Furthermore, fMRI can be useful for assessing memory reorganization processes in TLE patients [27]. In line with findings of a previous study by Jokeit et al. [7], we demonstrated greater fMRI activations in mesiotemporal regions contralateral to the seizure onset zone in patients with (left and right) TLE. Compared to findings in healthy controls, who demonstrated symmetrical, bilateral mesiotemporal activations during the task, this is most likely due to the underlying pathology or even indicates reorganization processes [7] within temporal or to extratemporal regions such as the insular cortex.

Most of the previous studies focused on reorganization processes in lesion-induced TLE. Very few descriptions exist about memory reorganization in subjects with non-lesional TLE [28]. In this study, subgroup analyses revealed that MRI negative left TLE patients showed bilateral activation patterns in mesiotemporal regions similar to those that have been described in healthy controls. Lateralization of the seizure onset zone in TLE patients has extensively been investigated by means of EEG, neuroimaging, and proton magnetic resonance spectroscopy [29]. Good concordances of the results of the different modalities were observed in subjects with lesional TLE. However, this was not applicable to non-lesional TLE. Results of spectroscopy studies revealed integrity of mesiotemporal structures on the side of the seizure focus in MRI negative TLE [29]. This may also explain the presence of bilateral fMRI activation in the left-sided MRI negative TLE group. However, there are also task-based and resting-state fMRI studies showing a reduced blood oxygenation level dependency (BOLD) signal in non-lesional TLE on the side of the seizure focus [28, 30]–presumably due to the ongoing epileptic activity. Compared to lesional TLE, however, non-lesional TLE patients showed less lateralized patterns of fMRI activations.

### Neurobiological and clinical implications

#### Functional reorganization within visuospatial memory networks

In left TLE patients, higher scoring during neuropsychological visuospatial memory testing was associated with greater fMRI activations exclusively in right>left mesiotemporal regions. Many descriptions indicate that, primarily, right mesiotemporal regions are involved in visual and spatial perception, which is in keeping with our findings [31, 32]. However, for the *Mosaik test*, significant correlations were found in the left parahippocampal gyrus, supporting the hypothesis that both left and right mesiotemporal structures are involved in visuospatial memory processing [33].

In right TLE, we did not observe significant activations in mesiotemporal structures. However, higher scoring during the *LPS-*, *LGT-3-*, and *Mosaik test* was associated with greater fMRI activations in the left insular cortex. Additionally, better performance during the *Mosaik test* was associated with activations in the right insular cortex and the right frontal operculum. The insular region has been associated with many different functions, such as cognition, attention, and visual and motor control [34, 35]. We suggest that damage to key structures in the right mesiotemporal lobe triggers reorganization processes, leading to activation of insular and opercular regions during visuospatial processing. The insular cortex has a key role during various cognitive tasks and shows multiple and complex connections to different brain areas [36]. Especially the left insular cortex is thought to be involved in several brain networks and might serve as a junction for different information [37]. This could explain our findings of insular involvement in visuospatial memory functions, since the insular cortex might support right mesiotemporal structures after damage. Lee et al. demonstrated that visuospatial memory performance did not differ before and after right-sided mesiotemporal surgical intervention in right TLE patients [38], which might point to the existence of multiple extratemporal systems supporting visuospatial memory function. However, further studies are needed in order to confirm our findings and to clarify the role of insular regions in visuospatial memory function.

#### Impairment of visuospatial memory networks and the DMN in TLE

In both, left and right TLE, mesiotemporal structures showed strong connectivity to contralateral and ipsilateral areas located within and outside the mesial temporal lobe. Overall, mesiotemporal regions in right TLE patients revealed reduced connectivity patterns compared to subjects with left TLE. This is in keeping with previous studies that showed that diminutions of limbic FC were more severe in right-sided than left-sided TLE patients [11, 39].

Reduced connectivity between left temporal regions and frontal areas has been described in left TLE and has been proposed to be associated with memory loss and language impairment [39]. However, it has also been speculated that the reduction of FC between the diseased temporal lobe and frontal areas might help in preserving cognitive functions, in terms of a memory protection mechanism by downregulation of a devastated network [11]. In our study, strong connectivity was observed between right mesiotemporal structures and frontal areas in left TLE, possibly indicating a compensatory upregulation of the contralateral memory network.

In left TLE, strong connectivity was also detected between the left insula and right temporal lobe areas, which was similarly observed in right TLE with the left insula being strongly connected to left temporal areas, underlining the driving role of left insular regions for visuospatial memory function. The connectivity pattern between the right insula and temporal regions was almost similar in right and left TLE patients, underpinning the integration of insular regions within visuospatial memory networks.

The FC subgroup analysis in lesional left and right TLE revealed comparable results to the main analysis. However, interestingly, non-lesional left TLE patients showed stronger connectivity within right hemispheric areas. Thus, although these patients demonstrate more bilateral/ipsilateral fMRI activation patterns during the RHWT, as opposed to lesion-induced TLE, the underlying visuospatial memory network exhibits a more rightward pattern. These findings underscore the complexitiy of memory reorganization processes in MRI negative TLE patients [28].

Using ICA, decreased involvement of mesiotemporal structures, particularly of hippocampal regions ipsilateral to the seizure onset zone into the DMN was revealed. The DMN is considered to decrease its activity during the active performance of a task [14]. Nonetheless, there is also evidence that the DMN serves as an active component during task execution [40] and, therefore, also may have a significant role as a part of memory-related brain networks [14]. Our findings are in keeping with previous findings in the literature [41] underlining the potential role of the DMN during active memory processing, showing reduced integration of right>left mesiotemporal structures into the DMN [40, 41]. FC changes appear to have a negative impact on a variety of cognitive and social skills, especially in right-sided TLE. As demonstrated in a previous study, it could be shown that disruptions of right limbic networks, predominantly detectable in right TLE, interfere with social-cognitive abilities [42].

In non-lesional TLE, there was no (para)hippocampal integration during task execution, which might indicate differences in mesiotemporal DMN component downregulation/upregulation mechanisms during cognitive processing in these patients as compared to subjects with lesion-induced TLE. Thus, our data may suggest differences in DMN involvement during active task execution in lesional (left/right) and MRI negative TLE. Nonetheless, further studies are needed to clarify the role of the DMN during active cognitive processing in patients with TLE.

### Limitations

Our study has several limitations. No healthy controls were available for the current study, which prohibited a direct comparison between TLE patients and healthy subjects. However, information regarding activation patterns in healthy subjects in this study was based on data previously described by Jokeit et al. [7].

Due to the retrospective nature of the study, the overall number of patients investigated with the RHWT was relatively small with different underlying pathologies and differences in disease duration. This has been addressed by dividing patients into subgroups with lesional and non-lesional left or right TLE. Further studies will be needed to define the role of epilepsy-related variables, such as underlying pathology, lesion localization, etc. on the organization of memory function.

In this study, we report findings based on uncorrected p-values, because the small sample influences statistical power and renders multiple comparison correction too strict. Although FWE/FDR correction is pivotal to reduce the occurrence of type I error, there is evidence of an increased type II error rate contemporaneously, primarily, with respect to modest samples [43]. Where applicable, we report uncorrected results with a conservative threshold (p<0.001), entailing caution in the interpretation of our findings. The small sample and the absence of healthy controls limited an SPM-based/CONN-based between group-comparison and the possibility to detail pathology-related fMRI activation/FC differences. However, a qualitative approach was used to detect fMRI activation-based/FC-based between-group differences.

Neuropsychological data was not available in all our patients. Therefore, correlational analyses were performed only in a subgroup of patients with lesional TLE. Findings will need to be confirmed in larger patient samples and further studies are underway, in order to address reorganization mechanisms of verbal and visual memory functions in larger patient cohorts with heterogeneous pathologies.

## Conclusion

Memory fMRI paradigms using adapted versions of the RHWT can be used for the assessment of visuospatial memory processing and its reorganization in TLE. While TLE in general leads to asymmetrical mesiotemporal activation, lesion-induced and non-lesional TLE patients reveal different fMRI activation patterns during the performance of the task. Furthermore, our data indicate that within the visual memory network insular regions serve as supporting brain areas in case of right mesiotemporal damage. Based on the data presented in this study, one can conclude that functional memory networks differ in right and left TLE.

## Supporting information

S1 Table Neuropsychological data. (DOCX)

S1 Text ROI to ROI: Left TLE. (TXT)

S2 Text ROI to ROI: Right TLE. (TXT)

S3 Text ROI to ROI: Lesional left TLE. (TXT)

S4 Text ROI to ROI: Lesional right TLE. (TXT)

S5 Text ROI to ROI: Non-lesional left TLE. (TXT)

## Abbreviations

DMN: Default mode network
EEG: Electroencephalography
FC: Functional connectivity
FDR: False discovery rate
FWE: Family-wise error
fMRI: Functional magnetic resonance imaging
ICA: Independent component analysis
RHWT: Roland’s Hometown Walking task
ROI: Region of interest
TLE: Temporal lobe epilepsy
