# Supplementary material for: Imaging visuospatial memory in temporal lobe epilepsy—Results of an fMRI study
Source: PLoS One. 2022 Feb 22;17(2):e0264349. doi: 10.1371/journal.pone.0264349 (PMC8863287; doi:10.1371/journal.pone.0264349)
Supplement: S1 Table — (DOCX) [file pone.0264349.s001.docx]

**Supplementary Table 1:** Neuropsychological data

| **Patient** | ***Mosaik test*** | ***Maze test*** | ***LGT-3 test*** | ***LPS-7 test*** | **Seizure Onset*** |
| --- | --- | --- | --- | --- | --- |
| 1 | + | + | – | + | R |
| 2 | + | + | + | + | R |
| 4 | + | – | + | + | R |
| 5 | + | + | + | + | R |
| 6 | + | – | + | + | R |
| 7 | + | + | + | + | R |
| 8 | + | + | – | + | L |
| 9 | + | + | + | + | L |
| 10 | + | + | + | + | L |
| 12 | + | + | + | – | L |
| 14 | + | + | + | + | L |
| 24 | + | + | + | + | L |

* Based on video-EEG monitoring and EEG findings

Seizure Onset: Hemisphere in which the seizure is generated

+: Data available

–: Data not available

L: Left

R: Right
